# Total Glycosides of *Cistanche deserticola* Promote Neurological Function Recovery by Inducing Neurovascular Regeneration *via* Nrf-2/Keap-1 Pathway in MCAO/R Rats

**DOI:** 10.3389/fphar.2020.00236

**Published:** 2020-03-17

**Authors:** Fujiang Wang, Ruiyan Li, Pengfei Tu, Jianping Chen, Kewu Zeng, Yong Jiang

**Affiliations:** ^1^ State Key Laboratory of Natural and Biomimetic Drugs, School of Pharmaceutical Sciences, Peking University, Beijing, China; ^2^ Department of Pharmacology, Changzhi Medical College, Shanxi, China; ^3^ School of Chinese Medicine, The University of Hong Kong, Hong Kong, Hong Kong

**Keywords:** *Cistanche deserticola*, cerebral injury, total glycosides, polysaccharides, oligosaccharides, Nrf-2/Keap-1 pathway

## Abstract

**Background:**

The traditional Chinese medicine *Cistanche deserticola* has been reported to be valid for cardiovascular and cerebrovascular diseases. However, its active components for the protection of ischemic stroke are not clear. We aimed to explore the active components of *C. deserticola* against ischemic stroke as well as its potential mechanisms.

**Methods:**

We investigated the brain protective effects of extracts from *C. deserticola*, total glycosides (TGs), polysaccharides (PSs), and oligosaccharides (OSs) in a rat model of middle cerebral artery occlusion-reperfusion (MCAO/R). 2, 3, 5-Triphenyltetrazolium chloride (TTC) staining was used to assess the cerebral infarction volume, and Evans blue assay was adopted to assess the blood-brain barrier (BBB) permeability. Then, the expressions CD31, α-SMA, PDGFRβ, SYN, PSD95, MAP-2, ZO-1, claudin-5, occludin, Keap-1, and Nrf-2 were analyzed using western blotting or immunofluorescence, and the activities MDA, SOD, CAT, and GSH-Px were analyzed using kits.

**Results:**

TGs treatment remarkably decreased neurological deficit scores and infarction volumes, promoted angiogenesis and neural remodeling, and effectively maintained blood-brain-barrier integrity compared with the model group. Furthermore, TGs significantly decreased MDA levels and increased antioxidant activities (SOD, CAT, and GSH-Px) in brains. Meanwhile, TGs remarkably downregulated Keap-1 expression and facilitated Nrf-2 nuclear translocation. On the contrary, no protective effects were observed for PSs and OSs groups.

**Conclusion:**

TGs are the main active components of *C. deserticola* against MCAO/R-induced cerebral injury, and protection is mainly *via* the Nrf-2/Keap-1 pathway.

## Introduction

Strokes are considered to be a major cause of death and disability in the world (Donnan et al., 2008). Nearly 87% of all stroke cases are triggered by ischemic stroke (Ovbiagele and Nguyen-Huynh, 2011). Currently, the most effective agent and the only FDA-approved drug used for ischemic stroke treatment is recombinant tissue plasminogen activator. However, a large amount of stroke patients fail to respond to this drug, owing to its narrow therapeutic time window and a serious risk of hemorrhagic complications (Lee et al., 2012; Schellinger and Kohrmann, 2014). A major challenge of thrombolytic treatment is ischemia/reperfusion (I/R) injury, which is considered as a main cause of brain injury and function destruction. Reperfusion after cerebral ischemia increases the risk of brain hemorrhage, while leading to neurovascular injury and producing excessive reactive oxygen species (ROS) which damage the blood-brain barrier (Alluri et al., 2015). A number of studies have confirmed that the disruption of the BBB is a major cause of the pathogenesis of ischemic stroke (Cao et al., 2016b).

The BBB consists mainly of endothelial cells, pericytes, astrocytes, neurons, and the basement membranes. The core components of the BBB are cerebral microvascular endothelial cells that are joined by tight junctions, thus restricting exogenous molecules into the brain. The pathological alterations of tight junctions—particularly occludin, claudin-5, and zonula occludens-1 (ZO-1)—significantly affect the BBB function during an ischemic stroke, especially barrier permeability (Liu et al., 2014; Hu et al., 2018; Liu et al., 2019). During I/R periods, excessive ROS is one of the main factors leading to the direct damage of brain neurons (Ding et al., 2014). ROS overproduction leads to the degradation of certain junctions and BBB disruption, which results in exogenous molecules entering into the brain through the BBB, leading to brain damage aggravation (Cheon et al., 2016; Zhang Q. Y. et al., 2017). Therefore, protection of the BBB by anti-oxidants has been regarded as a potential way to prevent reperfusion injury.

Besides the breakdown of the BBB, I/R can result in neurovascular injury and neuronal death (Jung et al., 2010). During a stroke, increased neuronal cell death may result from oxidative stress (Chi et al., 2018), and numerous studies have shown that ROS aggravates stroke severity and neurological damage (Kondo et al., 1997; Crack et al., 2001; Crack et al., 2006). Although clinical trials have not got satisfactory results, neuroprotection is still a promising strategy for treatment of acute ischemic stroke (Moretti et al., 2015). Thus, finding effective neuroprotection drugs to treat strokes is a benefit for stroke patients.

Traditional Chinese medicine (TCM) takes measures to intervene against the body's internal imbalance (Gaire, 2018). Owing to the complex pathogenesis of ischemic strokes, the multifactorial effect of TCM and their active constituents play a critical role in the treatment of strokes. *Cistanche deserticola* Y. C. Ma, widespread in arid or semi-arid areas throughout Mongolia and Northwest China, has been a widely used TCM herb for the treatment of various diseases such as forgetfulness and depression for more than 1,000 years in China. Modern pharmacological studies indicated that the crude extracts from *C. deserticola* showed multiple pharmacological activities, such as enhancing learning and memory function, neuroprotection, enhancing immunity, antioxidant, anti-aging, and antifatigue effects (Ko and Leung, 2007; Wang et al., 2012; Li et al., 2015). Chemical analysis of *C. deserticola* showed that its main constitutes include phenylethanoid glycosides, iridoid glycosides, polysaccharides and oligosaccharides (Jiang and Tu, 2009). However, the active components of *C. deserticola* for brain protection are not very clear.

The neuroprotective property of *C. deserticola* implies its therapeutic potential in cognitive-related illness such as stroke and depression, as well as Alzheimer's disease (Wang et al., 2017). However, research on the impact of *C. deserticola* on strokes, including its active components and action mechanisms, is very limited. In the current work, we explored the protective effect of three extracts from *C. deserticola*, total glycosides (TGs, phenylethanoid glycosides, and other glycosides), polysaccharides (PSs), and oligosaccharides (OSs) on cerebral I/R injuries. Our findings may contribute to the accurate clinical application of *C. deserticola* and provide a candidate agent for ischemic stroke therapy.

## Materials and Methods

### Chemicals and Reagents

**Figure 1:**
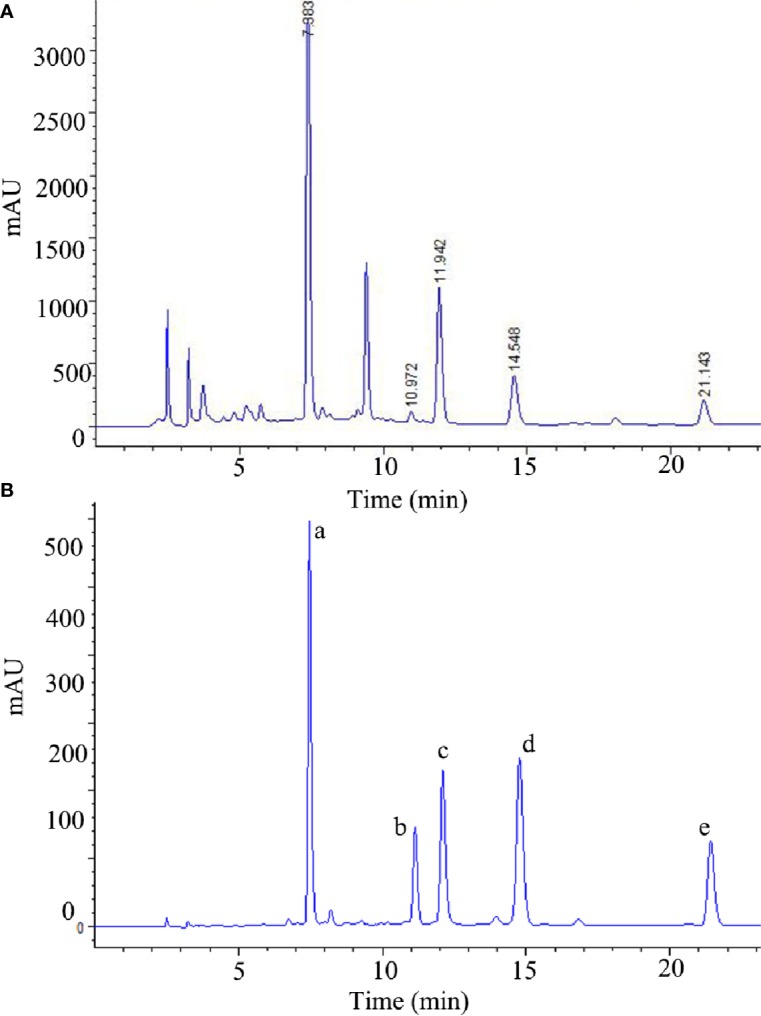
The HPLC chromatograms of the TGs **(A)** and standard references **(B)**. (a) echinacoside, (b) tubuloside A, (c) acteoside, (d) isoacteoside, (e) 2'-acetylacteoside of TGs.

The stems of *Cistanche deserticola* were purchased from Alashan, Inner Mongolia, and identified by one of the authors (P.-F. Tu). TGs, PSs, and OSs were prepared according to our previously reported method (Gao et al., 2015). Quantitative analysis of TGs was performed by high-performance liquid chromatography (HPLC) as previously described (Li et al, 2019), and its chromatogram is shown in **Figure 1**. The main components of TGs are echinacoside, tubuloside A, acteoside, isoacteoside, and 2'-acetylacteoside; their contents are 163.05 mg/g, 4.125 mg/g, 41.66 mg/g, 22.655 mg/g, and 12.045 mg/g, respectively. The contents of PSs and OSs are 69.42% and 65.24%, respectively, as determined by HPLC and the phenol–sulfuric acid analysis, respectively (Zhang A. et al., 2018; Shi et al, 2019).

The standard references of echinacoside (A0282), tubuloside A (A0942), acteoside (A0280), isoacteoside (A0281), and 2'-acetylacteoside (A0943) were purchased from Chengdu Must Biotechnology (Sichuan, China). The purities of all standards are more than 98%. Nissl stain H&E kits were bought from Boster (Wuhan, China). Edaravone (T0407-1) was bought from Target Mol (Shanghai, China). Rabbit anti-rat MAP-2 (ab32454), Nrf-2 (ab31163), PDGFRβ (ab32570), Keap-1 (ab66620), and mouse anti-rat CD31 (ab24590) were purchased from Abcam Inc (Cambridge, MA, USA). Rabbit anti-rat Claudin5 (BS1069), ZO-1 (BS9802M), and Occludin (BS72035) were bought from Bioworld Technology (Nanjing, China). Cell Signaling Technology Inc. (Boston, MA, USA) was the source of rabbit anti-rat Synapsin-1 (SYN,5297T), PSD95 (3450T), α-Smooth Muscle Actin (α-SMA,19245T). GAPDH (HRP-60004) was purchased from Proteintech Group, Inc. (Chicago, USA). Secondary antibodies were supplied by Zhongshan Golden Bridge Biotechnology (Beijing, China). Hoechst 33258 was obtained from Beyotime (Jiangsu, China).

### Animals

Sprague-Dawley rats (male, weighing 250–300g) were obtained from Vital River Laboratory Animal Technology (Beijing, China) and housed in an airconditioned room kept on a 12 h light/dark cycle. All animal experiments were performed in accordance with the animal research ARRIVE guidelines (Kilkenny et al., 2010; McGrath et al., 2010), and approved by the Institutional Animal Care and Use Committee of Peking University Health Science Center (LA2019123).

### Animal Experimental Protocols

The rats were subjected to MCAO/R, as previously described (Wang et al., 2018). Briefly, the left common carotid artery (CCA), external carotid artery (ECA), and internal carotid artery (ICA) were exposed, and a 3-0 nylon monofilament suture was inserted from the ECA into the ICA until reaching the middle cerebral artery (MCA). After 1.5 h of MCA occlusion, reperfusion was simulated by removing the filament. During the surgical procedure, the body temperature of all rats was maintained at 37.0°C.

### Drug Administration

The rats were randomly separated into six groups using the SPSS software version 22.0 as described (Jiang et al., 2014): normal group (NOR); model group (MOD); edaeavone group (positive drug, 6 mL/kg, EDI); TGs group (280 mg/kg, TGs); PSs group (280 mg/kg, PSs), and OSs group (280 mg/kg, OSs). TGs, PSs, and OSs were administrated ig once a day after MCAO/R for 14 days. The NOR and MOD groups were treated with normal saline. The animal numbers are shown in **Table 1**.

**Table 1:** Number of animals for different experimental groups and various parameters at 14 d after administration.

Parameters	NOR	MOD	EDI	TGs	PSs	OSs	Total
Weight, mNSS	18	18	18	18	18	18	108
TTC	8	8	8	8	8	8	48
H&E,Nissl stain	5	5	5	5	5	5	30
Immunofluorescent	5	5	5	5	5	5	30
Total	36	36	36	36	36	36	216

### Measurement of Weight and Modified Neurological Deficit Scores (mNSS)

Body weight was monitored on the 14th day using an ADVENTURE™ Digital Scale (OHAUS, New Jersey, USA). The mNSS was assessed according to the method described by FJ Wang (Wang et al., 2018), with minor revisions.

### 2, 3, 5-Triphenyltetrazolium Chloride (TTC) Staining

Infarct volume was measured as described previously (Wang et al., 2015). In brief, the brains were sectioned into seven equally spaced coronal blocks (2 mm). These sections were stained with 2% TTC (Coolaber, Beijing, China) at 37°C for 15 min. Infarct volume (%) = (ipsilateral ischemic hemisphere volume−contralateral ischemic hemisphere volume)/contralateral ischemic hemisphere volume × 100.

### Nissl and H&E Staining

The rats were deeply anesthetized, and the whole brain was then rapidly removed from the skull and fixed using 4% paraformaldehyde and embedded in paraffin wax and sectioned into slice of 7 µm thickness. The sections were stained with Nissl and H&E. In this study, six random 200 × 200 µm fields were captured in each tissue specimen with a light microscope. The number of Nissl's bodies was counted with IPP software version 6.0 (Media Cybernetics, Bethesda, USA).

### Evans Blue Assay

Rats were injected with 2% EB (Coolaber Science & Technology Co., LTD) after MCAO/R. Two hours later, the rats were anesthetized and the whole brain was then rapidly removed and homogenized in acetone. The supernatants were analyzed at 620 nm by an 800 TS absorbance reader (BioTek, USA).

### Measurement of the Activities of Catalase (CAT), Superoxide Dismutase (SOD), Malondialdehyde (MDA), and Glutathione Peroxidase (GSH-Px)

All serum samples were centrifuged at 4,000 × rpm for 15 min at 4°C, and then analyzed to detect the activities of MDA, CAT, SOD, and GSH-Px following the manufacturer's instructions (Jiangsu Meimian industrial Co., Ltd, China).

### Western Blotting Analysis

Brain tissues (100 mg) collected from each rat were homogenized and lysed in RIPA lysis buffer, and then analyzed to detect the protein concentration using a BCA kit (Beijing TransGen Biotech Co., Ltd.). Tissue total proteins were loaded on 10% SDS-PAGE gels and transferred onto a nitrocellulose membrane. The membrane was blocked using 5% skim milk, then incubated overnight with primary antibodies at 4°C. The membrane was then incubated with a secondary antibody. Western blot analysis was analyzed using Kodak Digital Imaging System (5200 Multi, Tanon, China).

### Immunofluorescent Analysis

Immunofluorescence staining for CD31, α-SMA, ZO-1, claudin5, occludin, PDGFRβ, SYN, PSD95, MAP-2, Nrf-2 and Keap-1 were performed. Primary antibodies against Nrf-2, CD31, α-SMA, ZO-1, claudin5, occludin, PDGFRβ, SYN, PSD95, MAP-2 and Keap-1 were diluted to 1:200 and 1:100, respectively. The secondary antibodys of Alexa Flur 488 mouse anti-rabbit IgG and rhodamine (TRITC) goat anti-rabbit IgG were both diluted to 1:200. The nucleuses were stained by Hoechst 33258. Images were captured using Vectra^®^ Polaris™ Automated Quantitative Pathology Imaging System (PerkinElmer, USA). The protein expression was analyzed using IPP software version 6.0.

### Statistical Analysis

All data were described as mean ± SD. SPSS software version 22.0 was performed for statistical analysis. One-way ANOVA was used when comparing different groups. *P* < 0.05 was considered to be statistical difference.

## Results

### TGs Increase Body Weight and Reduces Brain Damages in MCAO/R Rats

**Figure 2:**
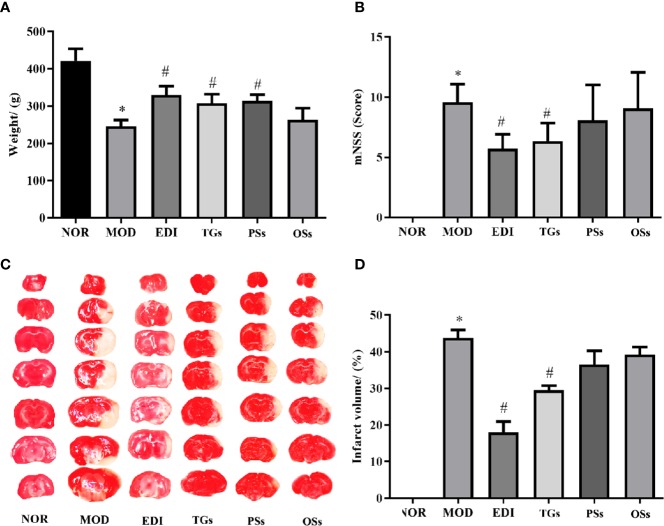
TGs reduce the brain damage induced by transient focal cerebral ischemia. **(A)** TGs increase weight in the MCAO/R rats. **(B)** TGs decrease neurological deficit scores at 14 d post-MCAO/R. **(C, D)** Representative photographs of TTC-stained coronal brain sections show viable (red) and dead (white) tissues; TTC staining shows that the cerebral infarction areas in the TGs group are significantly smaller than those in the model group. *P < 0.05 vs NOR. ^#^ *P* < 0.05 vs MOD.

After 14 days of cure with TGs, PSs, Oss, and EDI, the body weights, neurological deficits and infarct volumes of I/R rats were evaluated. The results showed that the body weights in the MOD group were greatly decreased, while the decreased weights in TGs, PSs and EDI groups were obviously increased (**Figure 2A**). Neurological deficit scores were substantially lowered by EDI and TGs (**Figure 2B**). The brain slices in NOR group rats were deep red and there were no infarctions, while the rats of the MOD group showed a large ipsilateral cerebral infarction. After TGs treatment, the infarct volumes were significantly reduced (**Figures 2C, D**). The PSs and OSs treatment showed no obvious effect on the above indexes. The above data showed that TGs could markedly alleviate the I/R-induced cerebral injury, but PSs and OSs could not.

### TGs Ameliorate Histopathological Damage in MCAO/R Rats

**Figure 3:**
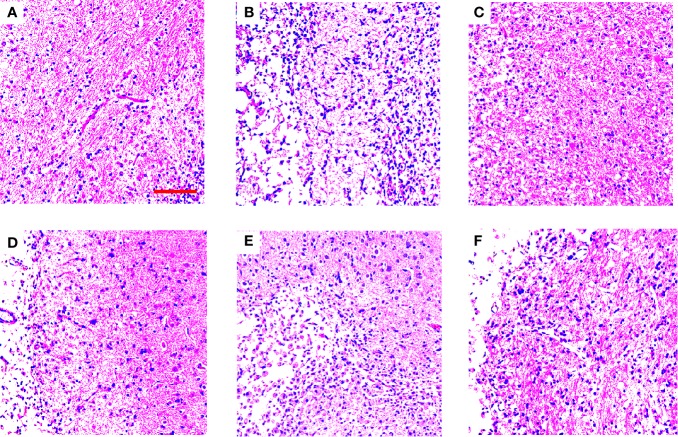
TGs ameliorate histopathological damage in MCAO/R rats. HE staining of coronal sections in the penumbra of ischemic areas of NOR, MOD, EDI, TGs, PSs, and OSs groups at 14 d after I/R. The cell morphology in the MOD group is shrinkage, nuclear pyknosis, and vacuolization, while the cells in the TGs group are relatively normal and show less damage, compared with those in the MOD group. **(A–F)** are NOR, MOD, EDI, TGs, PSs, and OSs groups, respectively. Scale bar = 100 µm.

In order to determine some of the effects of TGs, PSs, and OSs treatment on histopathological damages, H&E staining was done to reveal pathological damage. The histomorphological structures of brains in the NOR group were arranged regularly. The morphology changes in the TGs groups were slighter than those in the MOD group. However, the PSs and OSs treatment groups showed no significant amelioration of the morphology changes (**Figure 3**).

### TGs Attenuate Neuronal Injury After I/R-Induced Rats

**Figure 4:**
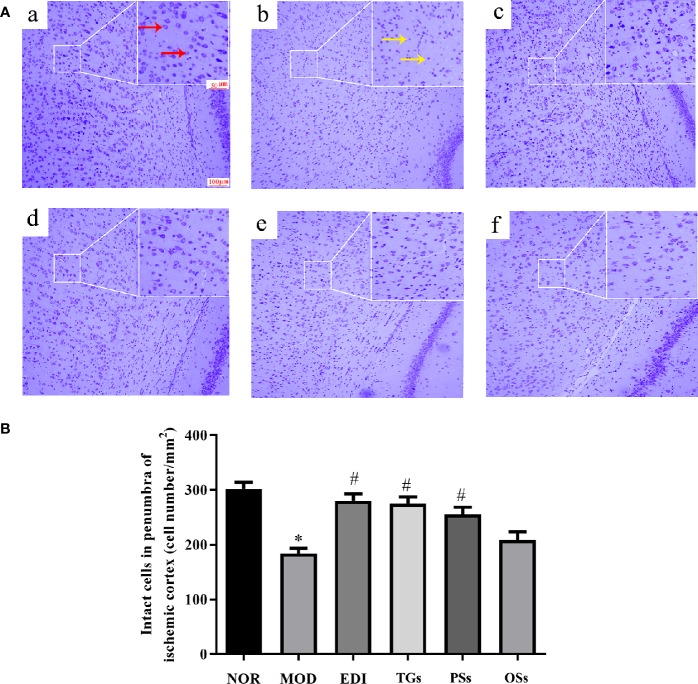
TGs attenuate neuronal injury after I/R-treated rats. **(A)** Nissl staining in the penumbra of ischemic area of I/R rats. Normal neurons have normal morphology with clear nucleolus, abundant cytoplasm, and intact structure (red arrow). Abnormal neurons are shrunken and deep stained (yellow arrow), while a greater increase of intact cells is evident in the TGs group compared with the MOD group. a–f are NOR, MOD, EDI, TGs, PSs and OSs groups, respectively. **(B)** Quantitative analysis of intact cells in penumbra of ischemic area at 14 days after treatment. *P < 0.05 vs NOR, ^#^ *P* < 0.05 vs MOD.

Nissl staining showed the histopathological changes of neurons in the penumbra of ischemic area. As shown in **Figure 4**, the normal neurons had a clear nucleolus and intact structure. In the MOD group, the neurons had enlarged intercellular spaces. The nissl bodies were disappeared, shrunken and deep stained. However, these changes were rarely observed in the EDI, TGs, and PSs groups. These results illustrated that TGs and PSs could significantly attenuate ischemia/reperfusion-induced neuronal injury.

### TGs Attenuate BBB Disruption After I/R-Treated Rats

**Figure 5:**
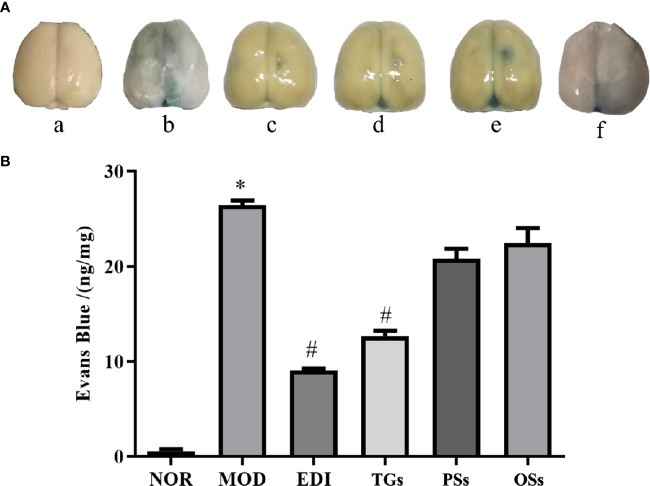
TGs attenuate BBB disruption after I/R injury. **(A)** Representative pictures of Evans blue extravasation. a–f are NOR, MOD, EDI, TGs, PSs, and OSs groups, respectively. **(B)** Quantitative analysis of Evans blue extravasation from brain extracts at 14 days after treatment. *P < 0.05 vs NOR, ^#^ *P* < 0.05 vs MOD.

Evans blue assay is a classical method for researching the change of BBB permeability. The experiment results showed that increased Evans blue was observed in the MOD group, while there was significantly decreased Evans blue in the TGs and EDI treated rats. Moreover, there was no significant difference between PSs and OSs therapy groups (**Figure 5**). These results suggested that TGs could significantly attenuate BBB disruption.

### TGs Promote Angiogenesis in I/R Injured Rats

**Figure 6:**
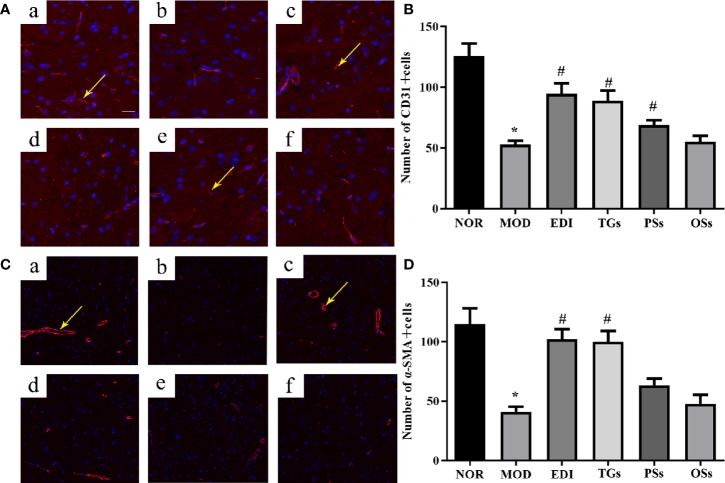
TGs promote neovascularization in rats with cerebral I/R. Representative images obtained from the cortex penumbra of ischemic hemispheres. **(A, C)** Representative immunoﬂuorescence images of CD31 and α-SMA in the I/R rats after treatment with TGs, PSs and OSs for 14 days, respectively. a–f are NOR, MOD, EDI, TGs, PSs, and OSs groups, respectively. **(B, D)** The bar graphs show the analysis of positive immunostaining of CD31 and α-SAM in the I/R rats at 14 days after treatment. *P < 0.05 vs NOR, ^#^ *P* < 0.05 vs MOD. Scale bar in all panels = 100 μm.

More recent studies show that angiogenesis plays a critical role in neurological functional recovery and prognostic outcome after acute ischemic stroke (Yuen et al., 2015). To evaluate effects of TGs, PSs, and OSs on angiogenesis, the CD31 and α-SMA were used to quantify the capillary numbers. Immunofluorescence staining showed that the MOD group caused a remarkable decrease in the expressions of CD31 (**Figures 6A**, **B**) and α-SMA (**Figures 6C**, **D**) in the penumbra of ischemic areas of I/R rats, in comparison with the normal rats. This result illustrated that I/R could cause the vascular damage in the cortex penumbra of ischemic hemispheres. However, the TGs and EDI treatment remarkably increased capillary density, angiogenesis, and arteriogenesis as indicated by increased expressions of CD31 and α-SMA. These results suggest that TGs could promote angiogenesis in the ischemic penumbra of I/R rats, but the PSs and OSs could not.

### TGs Increase Expression of Tight Junction Proteins in I/R Injured Rats

**Figure 7:**
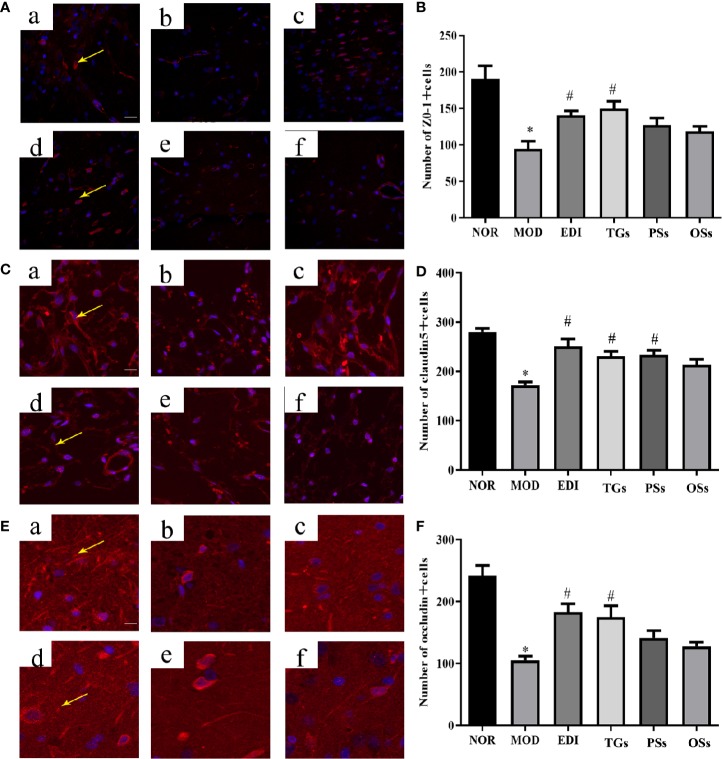
TGs increase expression of tight junction proteins in MCAO/R rats. Representative images obtained from the cortex penumbra of ischemic hemispheres. **(A, C, E)** Protein expression levels of ZO-1, claudin5, and occludin were measured by immunoﬂuorescence, respectively. **(B, D, F)** Statistical analysis of the numbers of ZO-1, claudin5, and occludin positive cells, respectively. a–f are NOR, MOD, EDI, TGs, PSs and OSs groups, respectively. *P < 0.05 vs NOR, ^#^ *P* < 0.05 vs MOD. Scale bar = 50 μm.

BBB disruption can elevate brain water content and tissue swelling, leading to brain injury. Tight junction proteins are important structural components of the BBB (Tenreiro et al., 2016; Jiang et al., 2018). To test whether TGs, PSs, and OSs treatment after stroke might influence BBB integrity, the expressions of ZO-1, claudin-5, and occludin were performed by immunoﬂuorescence analysis. The results indicated that the expressions of claudin-5, occludin, and ZO-1 were visibly decreased in the MOD group. However, they were substantially increased after 14 days of TGs administration. PSs and OSs groups showed no significant changes in these protein expressions (**Figure 7**). These data indicated that the TGs can regulate tight junction protein expressions and probably maintain BBB integrity after I/R injury.

### TGs Increase Pericyte Coverage on Capillaries in I/R Injured Rats

**Figure 8:**
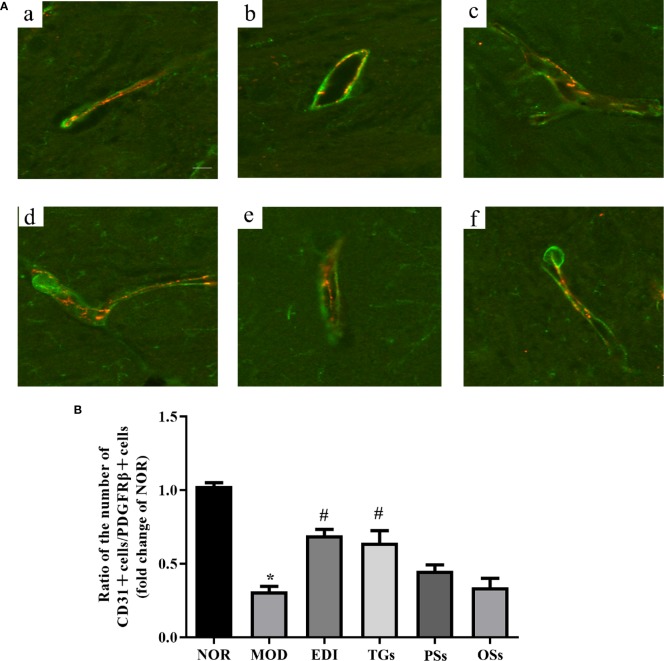
TGs increases pericyte coverage on capillaries in I/R injury rats. Representative images obtained from the ischemic penumbra of cortex. **(A)** Representative immunoﬂuorescence images of PDGFRβ (green) and CD31 (red) staining in the MCAO/R rats at 14 days after TGs, PSs and OSs treatment. a–f are NOR, MOD, EDI, TGs, PSs and OSs groups, respectively. **(B)** Ratio of CD31-positive cell numbers to the PDGFRβ-positive cell numbers in each tissue. *P < 0.05 vs NOR, ^#^ *P* < 0.05 vs MOD. Scale bar in all panels = 20 μm.

Pericyte coverage on capillaries plays a critical role in maintaining BBB integrity (Armulik et al., 2010; Daneman et al., 2010). Thus, we tested whether pericyte coverage could be increased by TGs, PSs, and OSs treatment. Immunoﬂuorescence intensity analysis results showed that both PDGFRβ and CD31 expressions were dramatically decreased in the MOD group. Administration of TGs to the I/R rats significantly recovered or even increased the expression intensities of the PDGFRβ and CD31, but no difference was observed in the PSs and OSs treatment groups (**Figure 8**). Thus, treatment of TGs could significantly increase pericyte coverage. These findings further confirmed that TGs can maintain the integrity of BBB after I/R.

### TGs Promote Neural Remodeling in I/R Injured Rats

**Figure 9:**
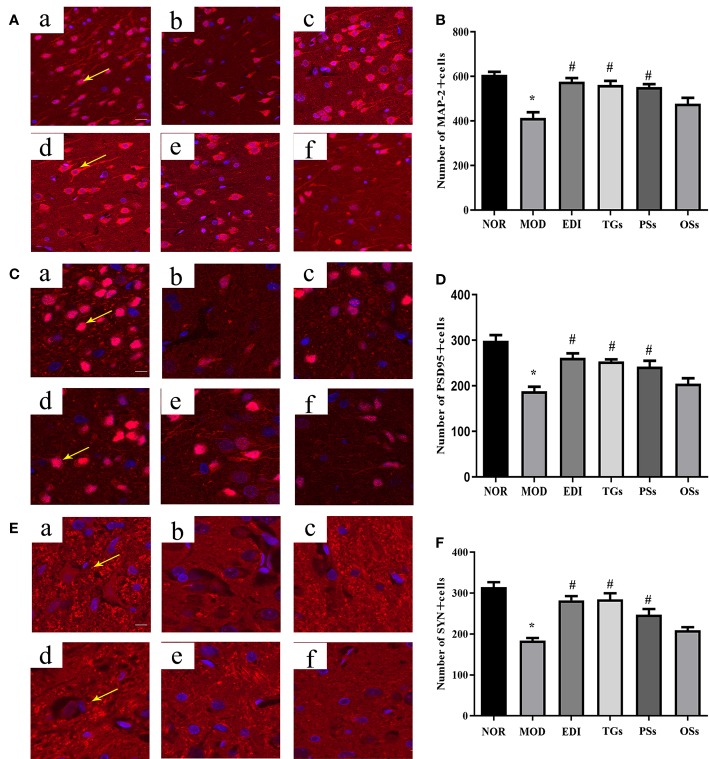
TGs promote neural remodeling in I/R injury rats. Representative images obtained from the cortex penumbra of ischemic hemispheres. **(A, C, E)** Representative immunoﬂuorescence images of MAP-2, PSD95 and SYN in the MCAO/R rats at 14 days after TGs, PSs and OSs treatment. **(B, D, F)** Statistical analysis of the numbers of MAP-2, PSD95 and SYN positive cells, respectively. a–f are NOR, MOD, EDI, TGs, PSs and OSs groups, respectively. *P < 0.05 vs NOR, ^#^ *P* < 0.05 vs MOD. Scale bar in all panels = 50 μm.

**Figure 10:**
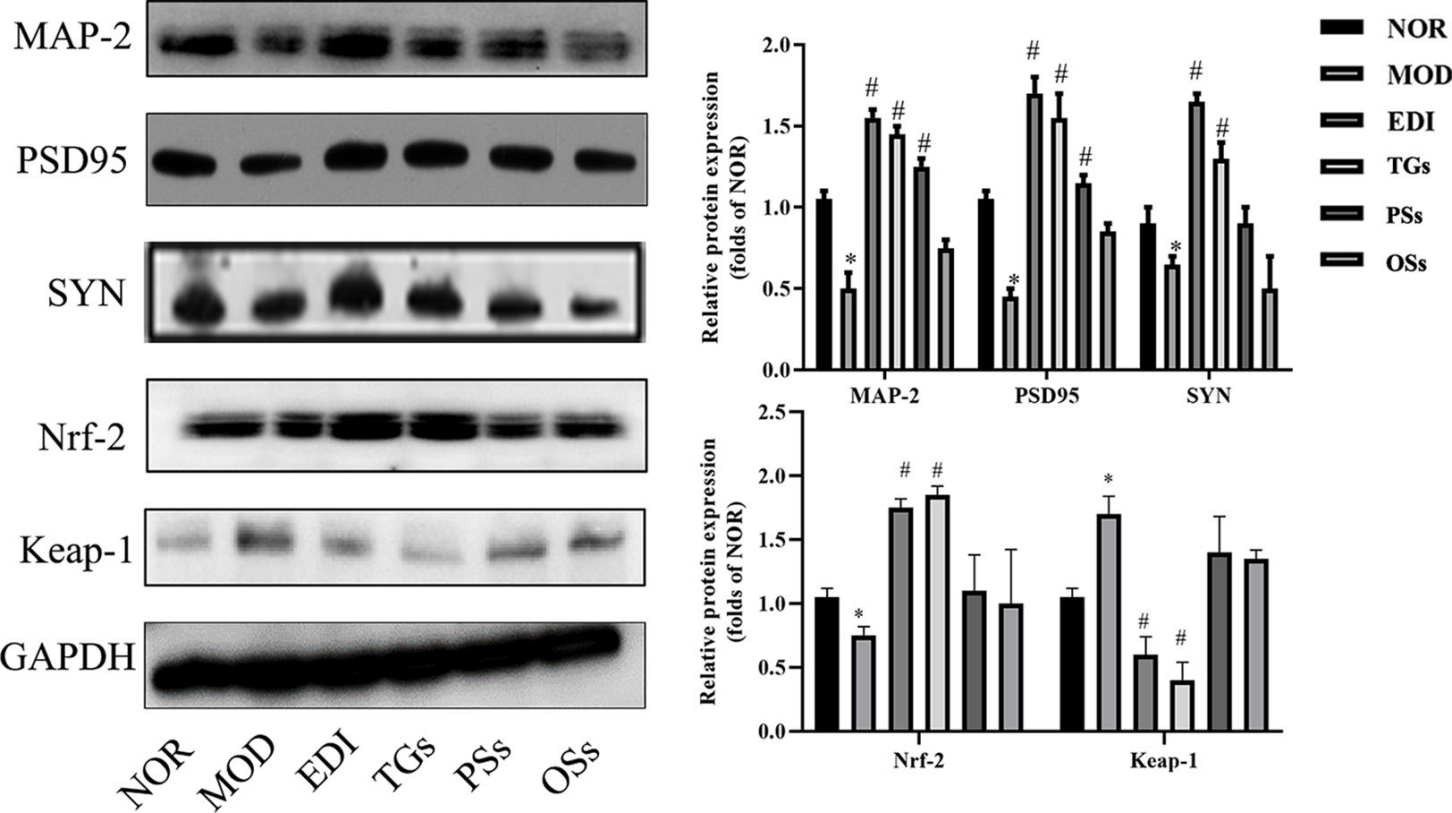
TGs promote the expressions of MAP-2, PSD95, SYN and Nrf-2 and reduce the expression of Keap-1 in I/R-treated rats. *P < 0.05 vs NOR, ^#^ *P* < 0.05 vs MOD.

According to numerous studies, neurogenesis after a stroke can significantly improve functional recovery (Grefkes and Ward, 2014; Zhang et al., 2019). Synaptophysin (SYN), postsynaptic density 95 (PSD-95) proteins and microtubule-associated protein 2 (MAP-2) were used as markers to examine neuronal plasticity in the ischemic penumbra of the cortex. In order to assess the effects of TGs, PSs, and OSs treatment on neurogenesis in I/R injured rats, the immunoﬂuorescence and western blot for SYN, PSD95 and MAP-2 expressions were performed. As shown in **Figures 9** and **10**, the SYN, PSD95 and MAP-2 expression levels in I/R rats after 14 days reperfusion decreased in comparison with the NOR rats, while the TGs and PSs cure could significantly up-regulate their expression levels. The OSs group had no significant change compared with the MOD group. The data indicated that the TGs and PSs cure was able to dramatically promote neural remodeling after I/R injury.

### TGs Alter Nrf-2 and Keap-1 Expressions in I/R Injured Rats

**Figure 11:**
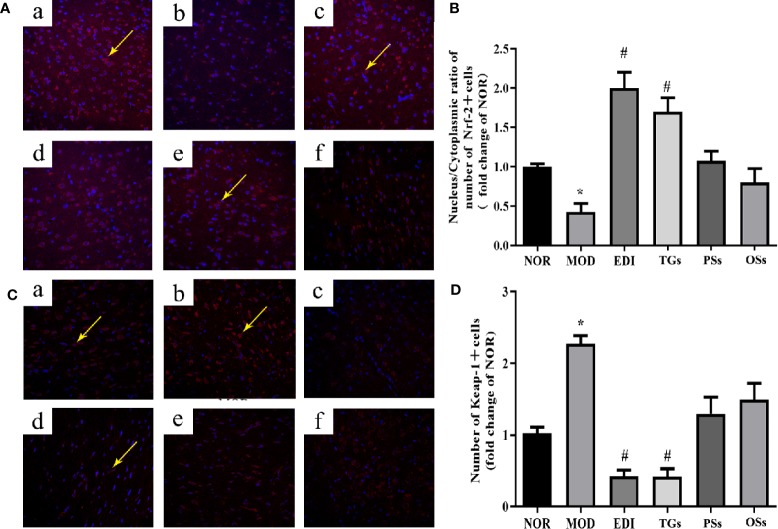
TGs significantly decrease the expression of Keap-1 and facilitate the nuclear translocation of Nrf-2. Representative images obtained from the cortex penumbraof ischemic hemispheres. **(A)** Keap-1 dependent Nrf-2 nuclear translocation was observed in I/R rats brain tissue. **(C)** Keap1 degradation after TGs treatment to I/R rats braintissue. The analysis was carried out using immunofluorescence staining. af are NOR, MOD, EDI, TGs, PSs and OSs groups, respectively. **(B**, **D)** Bar graphs show aquantification of the nucleus/cytoplasmic ratio of Nrf-2 and the expression of Keap-1. *P < 0.05 vs NOR, ^#^ *P* < 0.05 vs MOD. Scale bar = 100 mm.

Oxidative stress is a main pathogenic mechanism in I/R injury (Ya et al., 2018; Yu et al., 2018). The studies verified that Nrf-2 is a master regulator of antioxidative responses (Thompson et al., 2015). To investigate Nrf-2 and Keap-1 mediated oxidative responses after I/R injury, we evaluated the cytoplasmic expression as well as nuclear translocation of Keap-1. Meanwhile, the expression of Nrf-2 in I/R injured rats brain tissues was also assayed (**Figures 10** and **11**). According to the immunofluorescence analysis, Nrf-2 was found to be mainly located at the cytoplasm in the NOR group. In the TGs group, the expression of Nrf-2 in cytoplasmic localization was downregulated, but upregulated in the nucleus, and a decreased Keap-1 expression was also observed. The data showed that the brain protection of TGs could be associated with the modulation of Nrf-2 and Keap-1.

### TGs Attenuate Brain Tissue Oxidative Stress in I/R Injured Rats

**Figure 12:**
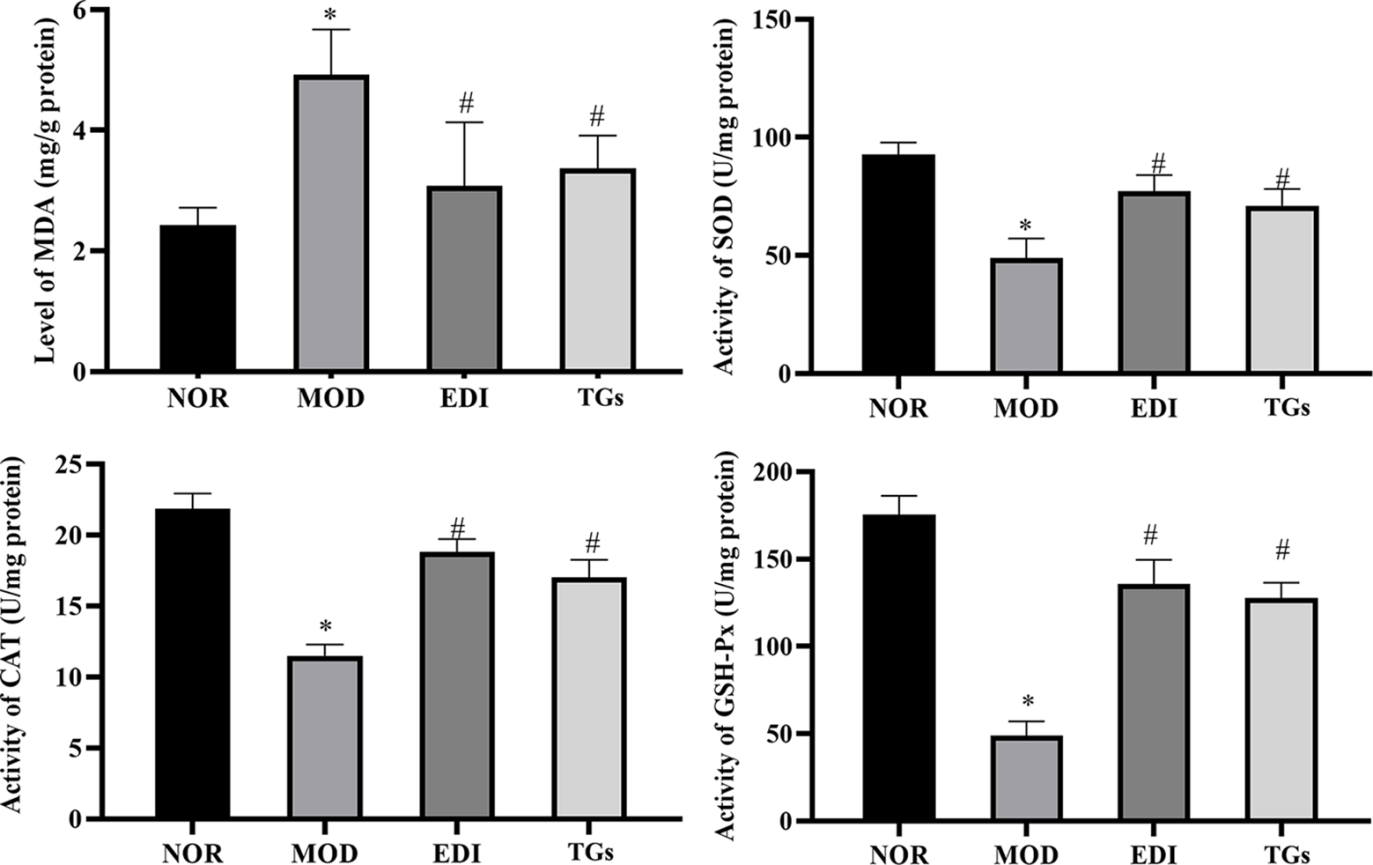
TGs elevate the levels of SOD, CAT and GSH-Px and reduces the level of MDA in I/R-treated rats. The content of MDA increases in the brain tissue after I/Rinjury, along with the activities of SOD, CAT, and GSH-Px decrease. TGs treatment reverses these injury-induced changes. *P < 0.05 vs NOR, ^#^ *P* < 0.05 vs MOD.

In order to confirm the antioxidative effects of TGs, the activities of SOD, CAT, GSH-Px and MDA were evaluated in I/R injured rats. In **Figure 12**, the content of MDA was markedly increased in the MOD group, and at the same time the activities of SOD, CAT, and GSH-Px were decreased, compared to the normal rats. Conversely, TGs treatment led to a significant decrease in the content of MDA and increase in the activities of SOD, CAT and GSH-Px. These results further confirmed the antioxidation activity of TGs.

## Discussion

Many studies suggest that the TCM *C. deserticola* has extensive biological activities, e.g. enhancing the ability to learn, memory, and immunity (Dong et al., 2007; Jiang and Tu, 2009; Wang et al., 2017; Xia et al., 2018). However, the active components of *C. deserticola* for neuroprotection remain unclear. The current work aims to screen the active components from *C. deserticola* against ischemic stroke on the MCAO/R model. Three extracts from *C. deserticola* (TGs, PSs, and OSs) were used to evaluate their effects on MCAO/R rats, as well as possible mechanisms.

Stroke is a common acute cerebrovascular disease. Epidemiological studies show that stroke is more common in men than in women (Sealy-Jefferson et al., 2012; Guzik and Bushnell, 2017). Thus, in our experiment, male rats were adopted for the tests. Our results proved that I/R induction accelerated oxidative stress and infarct volume, breaking the BBB and leading to nerve and cerebrovascular injury. After screening, TGs were found to decrease infarct volume and promote neural remodeling and angiogenesis. Moreover, TGs were observed to maintain BBB integrity after I/R injury. On the contrary, PSs and OSs bring no significant alleviation to I/R injury. Thus, TGs are considered the major active fraction of *C. deserticola* for neuroprotection, potentially through promoting neural remodeling, angiogenesis and BBB integrity *via* activating Nrf-2/Keap-1 pathway.

Mounting evidence indicates that establishment of effective collateral circulation is significantly important for avoiding the formation of infarction and ischemic penumbra, and is a critical treatment at an early stage of ischemic stroke (ElAli, 2016; Iwasawa et al., 2016). The proliferation of vascular endothelial cells and smooth muscle cells after ischemic infarction determines the establishment of collateral circulation. However, the ischemia models have a common phenomenon—that is, oxidative stress widely existed in the brain microvasculature. Study data have displayed that a large number of antioxidants can disturb the function of the BBB and the properties of angiogenesis (Mentor and Fisher, 2017). CD31 and α-SMA are the markers of vascular endothelial cells as well as smooth muscle cells, respectively (Saboor et al., 2016). To investigate the effect on the above-mentioned cell proliferation of the extracts from *C. deserticola*, we examined the expressions of CD31 and α-SMA in the cerebral ischemic penumbra homogenate. Our data showed that TGs strikingly enhanced the expressions of CD31 and α-SMA. However, there was no significant differences for PSs and OSs groups. Therefore, we deduced that TGs may reduce brain damage by promoting angiogenesis *via* increasing the expressions of CD31 and α-SMA, whereas PSs and OSs provided no such protection from brain damage. These results further confirmed that only TGs could prevent cerebral I/R injury.

Ischemic stroke can be thought as the result of cerebral ischemia caused by impairment in the neuronal plasticity or remodeling of brain areas. The majority of stroke patients suffer neurological deficits. Activating neurogenesis is a promising strategy for stroke patients to improve their neurological functions (Cramer and Chopp, 2000). Neurogenesis directly participates in neurological function recovery after brain I/R injury (Zhang et al., 2019). Previous research shows that TGs can improve the survival rate of hippocampal pyramidal cells and induce neurogenesis (Lian et al., 2017). Oxidative stress causes the loss of neurons during lots of diseases, such as Parkinson, stroke, and so on (Duan and Si, 2019; Singh et al., 2019). Nrf-2 transcribes lots of genes related to neuro-protection in their promoter region, mainly including SOD, MDA, CAT, and γ-glutamyl cysteine ligases, etc (Satoh et al., 2006). SYN, PSD-95, and MAP-2 proteins, which are associated closely with synaptic formation and neurotransmission, can be considered as markers of research neuronal plasticity in the ischemic penumbra region. After studying, we found that the cure with TGs could significantly increase the expressions of PSD95, SYN, and MAP-2, indicating that the cerebral protection of TGs was correlated with the enhanced neuronal plasticity during I/R. However, it's a pity that there is no obvious difference for PSs as well as OSs groups. These results indicated that TGs could enhance neuroplasticity after cerebral I/R injury.

Imaging research into stroke patients showed that BBB dysfunction can be thought as a striking attribute of the peri-ischemic brain (Bang et al., 2007). The TJs, which are composed of cytoplasmic proteins, transmembrane proteins, and junction adhesion molecules between capillary endothelial cells, are very important in maintaining BBB integrity (Ye et al., 2019). Among them, ZO-1, claudin-5, and occludin are the most important proteins in TJs. Mounting evidence indicates that the increased permeability of BBB induced by ischemia generally correlates with the alterations of ZO-1, claudin-5, and occludin (Cao et al., 2016a; Page et al., 2016; Yu et al., 2017; Liu et al., 2018). In this work, the results demonstrated that although TGs could significantly increase the expressions of ZO-1, claudin-5, and occludin proteins in MCAO-induced brain tissues, neither PSs nor OSs did. The BBB consists of cerebral endothelial cells and is closely associated with pericytes (Nyul-Toth et al., 2016). Pericytes are vital to BBB integrity (Bell et al., 2010). Ischemic stroke triggers pericytes death and detachment from brain endothelial cells in the acute phase, thus destabilizing the microvasculature and altering BBB properties (Zechariah et al., 2013). Our data showed that TGs could increase pericyte coverage on capillaries and increase the expression levels of ZO-1, claudin-5 and occludin. These phenomena proved that TGs could effectively protect BBB integrity after cerebral I/R injury. In summary, TGs may attenuate cerebral injury in multiple ways, such as promoting angiogenesis, improving neuronal plasticity, and maintaining the integrity of the BBB.

We then investigated the signaling pathway to explore the mechanism underlying the TGs brain protection. The process of I/R injury is multifactorial, and thus numerous mechanisms are involved in the pathogenesis. Oxidative stress is a fundamental risk factor contributing to I/R-induced brain injury (Suda et al., 2013), such as BBB structure damage, vascular endothelial dysfunction, and aggravation of ischemic neuronal injury (Xiong et al., 2015; Caglayan et al., 2019; Priestley et al., 2019). Thus, oxidative stress has become an attractive therapeutic target in I/R-induced brain injury. Phase 2 enzymes, which are mediated by nuclear factor E2-related factor-2 (Nrf-2), have been considered as an important means by which neurons protect themselves against oxidative stress (Suzuki and Yamamoto, 2015; Ya et al., 2018). Mounting evidences indicate that activation of Nrf-2 during I/R is a potential therapeutic target for neuroprotection (Ding et al., 2015; Zhang R. et al., 2017). Nrf-2, as an important regulator of endogenous antioxidant defense, mediates the level of heme oxygenase 1 (HO-1) and other antioxidant enzymes, such as NAD(P)H quinone oxidoreductase 1 (NQO1), SOD, CAT, GSH, and MDA (Siow et al., 2007; Ding et al., 2014). Moreover, Nrf-2 plays an important regulator role in angiogenesis. The present study shows that Nrf-2 can be significantly enhanced and activated in the process of vascular development (Wei et al., 2013).

As previously described (Jiang and Tu, 2009), TGs contain lots of bioactive compounds, for example echinacoside, tubuloside A, acteoside, isoacteoside and 2'-acetylacteoside, and some of them showed neuroprotective functions after cerebral I/R injury (Peng et al., 2016). Echinacoside has lots of pharmacological effects, such as antioxidation, anti-senescence, neuroprotection, anti-inflammation, promotion of cicatrization, hepatoprotection, promotion of bone formation and anti-tumor activities (Yu et al., 2016; Li et al., 2018; Zhang Y. et al., 2018; Ji et al., 2019; Xu et al., 2019). Recently, echinacoside has been identified as a potent antioxidant in the central nervous system (Lu et al., 2016). Echinacoside can cut down MDA content and improve the activities of SOD and GSH-Px in ischemia brain injury, and molecular docking analysis displayed that echinacoside may bind to Keap-1, leading to the Nrf-2 nuclear translocation (Li et al., 2018). The study of Xia showed that acteoside could decrease the infarct volume and brain water content to improve neurological deficits in MCAO/R rats through mitigating oxidative stress (Xia et al., 2018). Other studies have demonstrated that isoacteoside could increase the activities of cellular antioxidant enzymes, SOD and CAT in H_2_O_2_-treated V79-4 cells (Chae et al., 2005). Based on the above reports of the active compounds contained in TGs, it is possible to deduce that TGs could protect against ischemic stroke *via* the antioxidation pathways.

Li reported on the neuroprotective effects of phenylethanoid glycosides (PhGs) on H_2_O_2_-induced apoptosis on PC12 cells *via* the Nrf2/ARE pathway (Li et al., 2018). These PhGs significantly suppressed by triggering Nrf2 nuclear translocation and increasing expressions of HO-1, NQO1, glutamate cysteine ligase-catalytic subunit (GCLC), and glutamate-cysteine ligase modifier subunit (GCLM) (Li et al., 2018; Gong et al., 2019). Therefore, these findings suggest that the Nrf-2/ARE pathway plays a crucial role in PhGs-mediated protective effects on neuronal cells. Similarly, in this study, we found that TGs could decrease the level of MDA and increase the levels of SOD, CAT, and GSH-Px in the I/R rats. Meanwhile, TGs could upregulate the Nrf2 expression in the nucleus, downregulate the corresponding expression in the cytoplasm, and significantly decrease the Keap-1 expression. Therefore, the Nrf-2/Keap-1 pathway may be involved in TGs-mediated neuroprotective effects. Further validation of this pathway would be performed *in vitro* cell culture with oxygen-glucose deprivation/reoxygenation injury models in the future. Moreover, *C. deserticola* extracts were administrated in our study for 14 days continuously. Since adult neurogenesis would affect the interpretation of neuroprotective effects during 14 days reperfusion, neurogenesis cannot be excluded in our current experiment design in exploring the neuroprotective effect of CTs. This is the limitation of our research.

In conclusion, it is the TGs from *C. deserticola* that can enhance angiogenesis and neurogenesis as well as maintain the integrity of the BBB in I/R injury rats, but not the PSs and OSs. The effects could be mediated by the activation of the Nrf-2/Keap-1 pathway.

## Data Availability Statement

The raw data supporting the conclusions of this article will be made available by the authors, without undue reservation, to any qualified researcher.

## Ethics Statement

This work was carried out according to the Guidelines for Animal Experiments of Peking University. The study protocols were approved by the Institutional Animal Care and Use Committee at the Peking University Health Science Center (LA2019123).

## Author Contributions

YJ, KZ, and PT designed the research. FW performed the research. FW and RL analyzed the data. FW, RL, and JC wrote the manuscript and HPLC analysis. JC, KZ, YJ, and PT revised the manuscript.

## Funding

This study was supported by the National Key Research and Development Project (2017YFC1702400, 2019YFC1711000), the National Natural Science Foundation of China (81773932), and the National Key Technology R&D Program “New Drug Innovation” of China (2018ZX09711001-008-003).

## Conflict of Interest

The authors declare that the research was conducted in the absence of any commercial or financial relationships that could be construed as a potential conflict of interest.

The handling editor declared a shared affiliation, though no other collaboration, with the authors KZ, YJ, at time of review.

## References

[B1] Alluri H. Anasooya Shaji C. Davis M. L. Tharakan B. (2015). Oxygen-glucose deprivation and reoxygenation as an in vitro ischemia-reperfusion injury model for studying blood-brain barrier dysfunction. J. Vis. Exp. 99, e52699. 10.3791/52699 PMC4542466 25992584

[B2] Armulik A. Genove G. Mae M. Nisancioglu M. H. Wallgard E. Niaudet C. (2010). Pericytes regulate the blood-brain barrier. Nature 468 (7323), 557–561. 10.1038/nature09522 20944627

[B3] Bang O. Y. Buck B. H. Saver J. L. Alger J. R. Yoon S. R. Starkman S. (2007). Prediction of hemorrhagic transformation after recanalization therapy using T2*-permeability magnetic resonance imaging. Ann. Neurol. 62 (2), 170–176. 10.1002/ana.21174 17683090

[B4] Bell R. D. Winkler E. A. Sagare A. P. Singh I. LaRue B. Deane R. (2010). Pericytes control key neurovascular functions and neuronal phenotype in the adult brain and during brain aging. Neuron 68 (3), 409–427. 10.1016/j.neuron.2010.09.043 21040844 PMC3056408

[B5] Caglayan B. Kilic E. Dalay A. Altunay S. Tuzcu M. Erten F. (2019). Allyl isothiocyanate attenuates oxidative stress and inflammation by modulating Nrf2/HO-1 and NF-kappaB pathways in traumatic brain injury in mice. Mol. Biol. Rep. 46 (1), 241–250. 10.1007/s11033-018-4465-4 30406889

[B6] Cao G. Jiang N. Hu Y. Zhang Y. Wang G. Yin M. (2016a). Ruscogenin attenuates cerebral ischemia-induced blood-brain barrier dysfunction by suppressing TXNIP/NLRP3 inflammasome activation and the MAPK pathway. Int. J. Mol. Sci. 17 (9), 1–17. 10.3390/ijms17091418 PMC5037697 27589720

[B7] Cao G. Ye X. Xu Y. Yin M. Chen H. Kou J. (2016b). YiQiFuMai powder injection ameliorates blood-brain barrier dysfunction and brain edema after focal cerebral ischemia-reperfusion injury in mice. Drug Des. Dev. Ther. 10, 315–325. 10.2147/dddt.S96818 PMC4716731 26834461

[B8] Chae S. Kim J. S. Kang K. A. Bu H. D. Lee Y. Seo Y. R. (2005). Antioxidant activity of isoacteoside from *Clerodendron trichotomum* . J. Toxicol. Environ. Health A 68 (5), 389–400. 10.1080/15287390590900750 15799629

[B9] Cheon S. Y. Cho K. J. Kim S. Y. Kam E. H. Lee J. E. Koo B. N. (2016). Blockade of apoptosis signal-regulating kinase 1 attenuates matrix metalloproteinase 9 activity in brain endothelial cells and the subsequent apoptosis in neurons after ischemic injury. Front. Cell Neurosci. 10, 213. 10.3389/fncel.2016.00213 27642277 PMC5009117

[B10] Chi H. Chang H. Y. Sang T. K. (2018). Neuronal cell death mechanisms in major neurodegenerative diseases. Int. J. Mol. Sci. 19 (10), 1–18. 10.3390/ijms19103082 PMC6213751 30304824

[B11] Crack P. J. Taylor J. M. Flentjar N. J. de Haan J. Hertzog P. Iannello R. C. (2001). Increased infarct size and exacerbated apoptosis in the glutathione peroxidase-1 (Gpx-1) knockout mouse brain in response to ischemia/reperfusion injury. J. Neurochem. 78 (6), 1389–1399. 10.1046/j.1471-4159.2001.00535.x 11579147

[B12] Crack P. J. Taylor J. M. Ali U. Mansell A. Hertzog P. J. (2006). Potential contribution of NF-kappaB in neuronal cell death in the glutathione peroxidase-1 knockout mouse in response to ischemia-reperfusion injury. Stroke 37 (6), 1533–1538. 10.1161/01.Str.0000221708.17159.64 16627788

[B13] Cramer S. C. Chopp M. (2000). Recovery recapitulates ontogeny. Trends Neurosci. 23 (6), 265–271. 10.1016/s0166-2236(00)01562-9 10838596

[B14] Daneman R. Zhou L. Kebede A. A. Barres B. A. (2010). Pericytes are required for blood-brain barrier integrity during embryogenesis. Nature 468 (7323), 562–566. 10.1038/nature09513 20944625 PMC3241506

[B15] Ding Y. Chen M. Wang M. Wang M. Zhang T. Park J. (2014). Neuroprotection by acetyl-11-keto-beta-Boswellic acid, in ischemic brain injury involves the Nrf2/HO-1 defense pathway. Sci. Rep. 4, 7002. 10.1038/srep07002 25384416 PMC4227012

[B16] Ding Y. Chen M. Wang M. Li Y. Wen A. (2015). Posttreatment with 11-keto-beta-boswellic acid ameliorates cerebral ischemia-reperfusion injury: Nrf2/HO-1 pathway as a potential mechanism. Mol. Neurobiol. 52 (3), 1430–1439. 10.1007/s12035-014-8929-9 25452227

[B17] Dong Q. Yao J. Fang J. N. Ding K. (2007). Structural characterization and immunological activity of two cold-water extractable polysaccharides from Cistanche deserticola Y. C. Ma. Carbohydr. Res. 342 (10), 1343–1349. 10.1016/j.carres.2007.03.017 17442280

[B18] Donnan G. A. Fisher M. Macleod M. Davis S. M. (2008). Stroke. Lancet 371 (9624), 1612–1623. 10.1016/s0140-6736(08)60694-7 18468545

[B19] Duan Q. Si E. (2019). MicroRNA-25 aggravates Abeta1-42-induced hippocampal neuron injury in Alzheimer's disease by downregulating KLF2 via the Nrf2 signaling pathway in a mouse model. J. Cell Biochem. 120 (9), 15891–15905. 10.1002/jcb.28861 31144355

[B20] ElAli A. (2016). The implication of neurovascular unit signaling in controlling the subtle balance between injury and repair following ischemic stroke. Neural Regener. Res. 11 (6), 914–915. 10.4103/1673-5374.184485 PMC4962584 27482215

[B21] Gaire B. P. (2018). Herbal medicine in ischemic stroke: challenges and prospective. Chin. J. Integr. Med. 24 (4), 243–246. 10.1007/s11655-018-2828-2 29696521

[B22] Gao Y. Jiang Y. Dai F. Han Z. Liu H. Bao Z. (2015). Study on laxative constituents in *Cistanche deserticola* Y. C. Ma. Mod. Chin. Med. 17 (04), 19–22+26. 10.13313/j.issn.1673-4890.2015.4.003

[B23] Gong X. Xu Y. Ren K. Bai X. Zhang C. Li M. (2019). Phenylethanoid glycosides from *Paraboea martinii* protect rat pheochromocytoma (PC12) cells from hydrogen peroxide-induced cell injury. Biosci. Biotechnol. Biochem. 83 (12), 2202–2212. 10.1080/09168451.2019.1654359 31409200

[B24] Grefkes C. Ward N. S. (2014). Cortical reorganization after stroke: how much and how functional? Neuroscientist 20 (1), 56–70. 10.1177/1073858413491147 23774218

[B25] Guzik A. Bushnell C. (2017). Stroke epidemiology and risk factor management. Continuum (Minneap Minn) 23 (1, Cerebrovascular Dis), 15–39. 10.1212/con.0000000000000416 28157742

[B26] Hu S. Wu Y. Zhao B. Hu H. Zhu B. Sun Z. (2018). *Panax notoginseng* Saponins protect cerebral microvascular endothelial cells against oxygen-glucose deprivation/reperfusion-induced barrier dysfunction via activation of PI3K/Akt/Nrf2 antioxidant signaling pathway. Molecules 23 (11), 1–17. 10.3390/molecules23112781 PMC6278530 30373188

[B27] Iwasawa E. Ichijo M. Ishibashi S. Yokota T. (2016). Acute development of collateral circulation and therapeutic prospects in ischemic stroke. Neural Regener. Res. 11 (3), 368–371. 10.4103/1673-5374.179033 PMC4828985 27127459

[B28] Ji S. Li S. Zhao X. Kang N. Cao K. Zhu Y. (2019). Protective role of phenylethanoid glycosides, Torenoside B and Savatiside A, in Alzheimer's disease. Exp. Ther. Med. 17 (5), 3755–3767. 10.3892/etm.2019.7355 30988761 PMC6447766

[B29] Jiang Y. Tu P. F. (2009). Analysis of chemical constituents in *Cistanche* species. J. Chromatogr. A 1216 (11), 1970–1979. 10.1016/j.chroma.2008.07.031 18691718

[B30] Jiang T. Yu J. T. Zhu X. C. Wang H. F. Tan M. S. Cao L. (2014). Acute metformin preconditioning confers neuroprotection against focal cerebral ischaemia by pre-activation of AMPK-dependent autophagy. Br. J. Pharmacol. 171 (13), 3146–3157. 10.1111/bph.12655 24611741 PMC4080970

[B31] Jiang X. Andjelkovic A. V. Zhu L. Yang T. Bennett M. V. L. Chen J. (2018). Blood-brain barrier dysfunction and recovery after ischemic stroke. Prog. Neurobiol. 163-164, 144–171. 10.1016/j.pneurobio.2017.10.001 28987927 PMC5886838

[B32] Jung J. E. Kim G. S. Chen H. Maier C. M. Narasimhan P. Song Y. S. (2010). Reperfusion and neurovascular dysfunction in stroke: from basic mechanisms to potential strategies for neuroprotection. Mol. Neurobiol. 41 (2-3), 172–179. 10.1007/s12035-010-8102-z 20157789 PMC2877155

[B33] Kilkenny C. Browne W. J. Cuthill I. C. Emerson M. Altman D. G. (2010). Improving bioscience research reporting: the ARRIVE guidelines for reporting animal research. PLoS Biol. 8 (6), e1000412. 10.1371/journal.pbio.1000412 20613859 PMC2893951

[B34] Ko K. M. Leung H. Y. (2007). Enhancement of ATP generation capacity, antioxidant activity and immunomodulatory activities by Chinese Yang and Yin tonifying herbs. Chin. Med. 2, 3. 10.1186/1749-8546-2-3 17386115 PMC1847515

[B35] Kondo T. Reaume A. G. Huang T. T. Carlson E. Murakami K. Chen S. F. (1997). Reduction of CuZn-superoxide dismutase activity exacerbates neuronal cell injury and edema formation after transient focal cerebral ischemia. J. Neurosci. 17 (11), 4180–4189. 10.1523/JNEUROSCI.17-11-04180 9151735 PMC6573543

[B36] Lee M. Saver J. L. Alger J. R. Hao Q. Starkman S. Ali L. K. (2012). Blood-brain barrier permeability derangements in posterior circulation ischemic stroke: frequency and relation to hemorrhagic transformation. J. Neurol. Sci. 313 (1-2), 142–146. 10.1016/j.jns.2011.08.048 21945462 PMC3254818

[B37] Li N. Wang J. Ma J. Gu Z. Jiang C. Yu L. (2015). Neuroprotective effects of cistanches herba therapy on patients with moderate alzheimer's disease. Evid Based Complement Altern. Med. 2015, 103985. 10.1155/2015/103985 PMC4576016 26435722

[B38] Li M. Xu T. Zhou F. Wang M. Song H. Xiao X. (2018). Neuroprotective effects of four phenylethanoid glycosides on H(2)O(2)-induced apoptosis on PC12 cells via the Nrf2/ARE pathway. Int. J. Mol. Sci. 19 (4), 1–17. 10.3390/ijms19041135 PMC5979387 29642608

[B39] Li R. Zhao M. Tu P. Jiang Y. (2019). Simultaneous determination of five phenylethanoid glycosides in Cistanches Herba using quantitative analysis of multi-components by single marker. J. Chin. Pharm. Sci. 28 (08), 537–546. 10.5246/jcps.2019.08.051

[B40] Lian J. Wang L. Zhao F. Lin S. Yan X. Jia J. (2017). Effects of glycosides of cistanche on synaptic morphological plasticity in senescence accelerated mice. J. Baotou Medl Coll. 33 (08), 78–80. 10.16833/j.cnki.jbmc.2017.08.036

[B41] Liu Y. Wang D. Wang H. Qu Y. Xiao X. Zhu Y. (2014). The protective effect of HET0016 on brain edema and blood-brain barrier dysfunction after cerebral ischemia/reperfusion. Brain Res. 1544, 45–53. 10.1016/j.brainres.2013.11.031 24316243

[B42] Liu P. Zhang R. Liu D. Wang J. Yuan C. Zhao X. (2018). Time-course investigation of blood-brain barrier permeability and tight junction protein changes in a rat model of permanent focal ischemia. J. Physiol. Sci. 68 (2), 121–127. 10.1007/s12576-016-0516-6 28078626 PMC10716957

[B43] Liu S. Chang L. Wei C. (2019). The sonic hedgehog pathway mediates Tongxinluo capsule-induced protection against blood-brain barrier disruption after ischaemic stroke in mice. Basic Clin. Pharmacol. Toxicol. 124 (6), 660–669. 10.1111/bcpt.13186 30548093

[B44] Lu C. W. Lin T. Y. Huang S. K. Wang S. J. (2016). Echinacoside inhibits glutamate release by suppressing voltage-dependent Ca(2+) entry and protein kinase C in rat cerebrocortical nerve terminals. Int. J. Mol. Sci. 17 (7), 1–13. 10.3390/ijms17071006 PMC4964382 27347934

[B45] McGrath J. C. Drummond G. B. McLachlan E. M. Kilkenny C. Wainwright C. L. (2010). Guidelines for reporting experiments involving animals: the ARRIVE guidelines. Br. J. Pharmacol. 160 (7), 1573–1576. 10.1111/j.1476-5381.2010.00873.x 20649560 PMC2936829

[B46] Mentor S. Fisher D. (2017). Aggressive antioxidant reductive stress impairs brain endothelial cell angiogenesis and blood brain barrier function. Curr. Neurovasc Res. 14 (1), 71–81. 10.2174/1567202613666161129113950 27897111

[B47] Moretti A. Ferrari F. Villa R. F. (2015). Neuroprotection for ischaemic stroke: current status and challenges. Pharmacol. Ther. 146, 23–34. 10.1016/j.pharmthera.2014.09.003 25196155

[B48] Nyul-Toth A. Suciu M. Molnar J. Fazakas C. Hasko J. Herman H. (2016). Differences in the molecular structure of the blood-brain barrier in the cerebral cortex and white matter: an in silico, in vitro, and ex vivo study. Am. J. Physiol. Heart Circ. Physiol. 310 (11), H1702–H1714. 10.1152/ajpheart.00774.2015 27059078

[B49] Ovbiagele B. Nguyen-Huynh M. N. (2011). Stroke epidemiology: advancing our understanding of disease mechanism and therapy. Neurotherapeutics 8 (3), 319–329. 10.1007/s13311-011-0053-1 21691873 PMC3250269

[B50] Page S. Munsell A. Al-Ahmad A. J. (2016). Cerebral hypoxia/ischemia selectively disrupts tight junctions complexes in stem cell-derived human brain microvascular endothelial cells. Fluids Barriers CNS 13 (1), 16. 10.1186/s12987-016-0042-1 27724968 PMC5057206

[B51] Peng F. Chen J. Wang X. Xu C. Liu T. Xu R. (2016). Changes in levels of phenylethanoid glycosides, antioxidant activity, and other quality traits in cistanche deserticola slices by steam processing. Chem. Pharm. Bull. (Tokyo) 64 (7), 1024–1030. 10.1248/cpb.c16-00033 27063326

[B52] Priestley J. R. C. Fink K. E. McCord J. M. Lombard J. H. (2019). NRF2 activation with protandim attenuates salt-induced vascular dysfunction and microvascular rarefaction. Microcirculation, 26 (7), e12575. 10.1111/micc.12575 31132190 PMC7154587

[B53] Saboor F. Reckmann A. N. Tomczyk C. U. Peters D. M. Weissmann N. Kaschtanow A. (2016). Nestin-expressing vascular wall cells drive development of pulmonary hypertension. Eur. Respir. J. 47 (3), 876–888. 10.1183/13993003.00574-2015 26699726 PMC5796529

[B54] Satoh T. Okamoto S. I. Cui J. Watanabe Y. Furuta K. Suzuki M. (2006). Activation of the Keap1/Nrf2 pathway for neuroprotection by electrophilic [correction of electrophillic] phase II inducers. Proc. Natl. Acad. Sci. U. S. A. 103 (3), 768–773. 10.1073/pnas.0505723102 16407140 PMC1334635

[B55] Schellinger P. D. Kohrmann M. (2014). 4.5-hour time window for intravenous thrombolysis with recombinant tissue-type plasminogen activator is established firmly. Stroke 45 (3), 912–913. 10.1161/strokeaha.113.002700 24526060

[B56] Sealy-Jefferson S. Wing J. J. Sanchez B. N. Brown D. L. Meurer W. J. Smith M. A. (2012). Age- and ethnic-specific sex differences in stroke risk. Gend Med. 9 (2), 121–128. 10.1016/j.genm.2012.02.002 22445684 PMC3481549

[B57] Shi Z. Wu Y. Zhu Y. Cui Wang M. Yin H. (2019). Quantitative determination of betaine, mannitol, fructose, glucose and sucrose in Cistanches Herba by HPLC-ELSD. Mod. Chin. Med. 32 (6), 1–11. 10.13313/j.issn.1673-4890.20190320006

[B58] Singh D. Reeta K. H. Sharma U. Jagannathan N. R. Dinda A. K. Gupta Y. K. (2019). Neuro-protective effect of monomethyl fumarate on ischemia reperfusion injury in rats: Role of Nrf2/HO1 pathway in peri-infarct region. Neurochem. Int. 126, 96–108. 10.1016/j.neuint.2019.03.010 30880045

[B59] Siow R. C. Ishii T. Mann G. E. (2007). Modulation of antioxidant gene expression by 4-hydroxynonenal: atheroprotective role of the Nrf2/ARE transcription pathway. Redox Rep. 12 (1), 11–15. 10.1179/135100007x162167 17263901

[B60] Suda S. Katsura K. Kanamaru T. Saito M. Katayama Y. (2013). Valproic acid attenuates ischemia-reperfusion injury in the rat brain through inhibition of oxidative stress and inflammation. Eur. J. Pharmacol. 707 (1-3), 26–31. 10.1016/j.ejphar.2013.03.020 23541723

[B61] Suzuki T. Yamamoto M. (2015). Molecular basis of the Keap1-Nrf2 system. Free Radic. Biol. Med. 88 (Pt B), 93–100. 10.1016/j.freeradbiomed.2015.06.006 26117331

[B62] Tenreiro M. M. Ferreira R. Bernardino L. Brito M. A. (2016). Cellular response of the blood-brain barrier to injury: Potential biomarkers and therapeutic targets for brain regeneration. Neurobiol. Dis. 91, 262–273. 10.1016/j.nbd.2016.03.014 26996728

[B63] Thompson J. W. Narayanan S. V. Koronowski K. B. Morris-Blanco K. Dave K. R. Perez-Pinzon M. A. (2015). Signaling pathways leading to ischemic mitochondrial neuroprotection. J. Bioenerg. Biomembr. 47 (1-2), 101–110. 10.1007/s10863-014-9574-8 25262285 PMC4861652

[B64] Wang T. Zhang X. Xie W. (2012). *Cistanche deserticola* Y. C. Ma, “Desert ginseng”: a review. Am. J. Chin. Med. 40 (6), 1123–1141. 10.1142/s0192415x12500838 23227786

[B65] Wang X. Wang S. Wang J. Guo H. Dong Z. Chai L. (2015). Neuroprotective effect of xueshuantong for injection (lyophilized) in transient and permanent rat cerebral ischemia model. Evid Based Complement Altern. Med. 2015, 134685. 10.1155/2015/134685 PMC4670871 26681963

[B66] Wang D. Wang H. Gu L. (2017). The antidepressant and cognitive improvement activities of the traditional chinese herb cistanche. Evid Based Complement Altern. Med. 2017, 3925903. 10.1155/2017/3925903 PMC5506466 28744316

[B67] Wang F. J. Wang S. X. Chai L. J. Zhang Y. Guo H. Hu L. M. (2018). Xueshuantong injection (lyophilized) combined with salvianolate lyophilized injection protects against focal cerebral ischemia/reperfusion injury in rats through attenuation of oxidative stress. Acta Pharmacol. Sin. 39 (6), 998–1011. 10.1038/aps.2017.128 29022576 PMC6256270

[B68] Wei Y. Gong J. Thimmulappa R. K. Kosmider B. Biswal S. Duh E. J. (2013). Nrf2 acts cell-autonomously in endothelium to regulate tip cell formation and vascular branching. Proc. Natl. Acad. Sci. U. S. A. 110 (41), E3910–E3918. 10.1073/pnas.1309276110 24062466 PMC3799376

[B69] Xia D. Zhang Z. Zhao Y. (2018). Acteoside attenuates oxidative stress and neuronal apoptosis in rats with focal cerebral ischemia-reperfusion injury. Biol. Pharm. Bull. 41 (11), 1645–1651. 10.1248/bpb.b18-00210 30381663

[B70] Xiong W. MacColl Garfinkel A. E. Li Y. Benowitz L. I. Cepko C. L. (2015). NRF2 promotes neuronal survival in neurodegeneration and acute nerve damage. J. Clin. Invest. 125 (4), 1433–1445. 10.1172/jci79735 25798616 PMC4396467

[B71] Xu H. T. Zhang C. G. He Y. Q. Shi S. S. Wang Y. L. Chou G. X. (2019). Phenylethanoid glycosides from the *Schnabelia nepetifolia* (Benth.) P.D.Cantino promote the proliferation of osteoblasts. Phytochemistry 164, 111–121. 10.1016/j.phytochem.2019.05.003 31125861

[B72] Ya B. L. Liu Q. Li H. F. Cheng H. J. Yu T. Chen L. (2018). Uric acid protects against focal cerebral ischemia/reperfusion-induced oxidative stress via activating Nrf2 and regulating neurotrophic factor expression. Oxid. Med. Cell Longev 2018, 6069150. 10.1155/2018/6069150 30581534 PMC6276484

[B73] Ye Z. Y. Xing H. Y. Wang B. Liu M. Lv P. Y. (2019). DL-3-n-butylphthalide protects the blood-brain barrier against ischemia/hypoxia injury via upregulation of tight junction proteins. Chin. Med. J. (Engl.) 132 (11), 1344–1353. 10.1097/cm9.0000000000000232 30939485 PMC6629356

[B74] Yu Q. Li X. Cao X. (2016). Cardioprotective effects of phenylethanoid glycoside-rich extract from *Cistanche deserticola* in ischemia-reperfusion-induced myocardial infarction in rats. Ann. Vasc. Surg. 34, 234–242. 10.1016/j.avsg.2016.04.002 27129809

[B75] Yu N. Wang Z. Chen Y. Yang J. Lu X. Guo Y. (2017). The ameliorative effect of bloodletting puncture at hand twelve Jing-well points on cerebral edema induced by permanent middle cerebral ischemia via protecting the tight junctions of the blood-brain barrier. BMC Complement Altern. Med. 17 (1), 470. 10.1186/s12906-017-1979-6 28950851 PMC5615481

[B76] Yu W. Gao D. Jin W. Liu S. Qi S. (2018). Propofol prevents oxidative stress by decreasing the ischemic accumulation of succinate in focal cerebral ischemia-reperfusion injury. Neurochem. Res. 43 (2), 420–429. 10.1007/s11064-017-2437-z 29168092

[B77] Yuen C. M. Chung S. Y. Tsai T. H. Sung P. H. Huang T. H. Chen Y. L. (2015). Extracorporeal shock wave effectively attenuates brain infarct volume and improves neurological function in rat after acute ischemic stroke. Am. J. Transl. Res. 7 (6), 976–994. 26279744 PMC4532733

[B78] Zechariah A. ElAli A. Doeppner T. R. Jin F. Hasan M. R. Helfrich I. (2013). Vascular endothelial growth factor promotes pericyte coverage of brain capillaries, improves cerebral blood flow during subsequent focal cerebral ischemia, and preserves the metabolic penumbra. Stroke 44 (6), 1690–1697. 10.1161/strokeaha.111.000240 23632977

[B79] Zhang Q. Y. Wang Z. J. Sun D. M. Wang Y. Xu P. Wu W. J. (2017). Novel therapeutic tffects of teonurine on ischemic stroke: new mechanisms of BBB integrity. Oxid. Med. Cell Longev 2017, 7150376. 10.1155/2017/7150376 28690765 PMC5485366

[B80] Zhang R. Xu M. Wang Y. Xie F. Zhang G. Qin X. (2017). Nrf2-a promising therapeutic target for defensing against oxidative stress in stroke. Mol. Neurobiol. 54 (8), 6006–6017. 10.1007/s12035-016-0111-0 27696223

[B81] Zhang A. Yang X. Li Q. Yang Y. Zhao G. Wang B. (2018). Immunostimulatory activity of water-extractable polysaccharides from *Cistanche deserticola* as a plant adjuvant in vitro and in vivo. PLoS One 13 (1), e0191356. 10.1371/journal.pone.0191356 29360858 PMC5779666

[B82] Zhang Y. Wang K. Chen H. He R. Cai R. Li J. (2018). Anti-inflammatory lignans and phenylethanoid glycosides from the root of *Isodon ternifolius* (D.Don) Kudo. Phytochemistry 153, 36–47. 10.1016/j.phytochem.2018.05.017 29860140

[B83] Zhang K. Zhang Q. Deng J. Li J. Li J. Wen L. (2019). ALK5 signaling pathway mediates neurogenesis and functional recovery after cerebral ischemia/reperfusion in rats via Gadd45b. Cell Death Dis. 10 (5), 360. 10.1038/s41419-019-1596-z 31043581 PMC6494915

